# Structure–function relationships in the Nab2 polyadenosine‐RNA binding Zn finger protein family

**DOI:** 10.1002/pro.3565

**Published:** 2019-01-16

**Authors:** Milo B. Fasken, Anita H. Corbett, Murray Stewart

**Affiliations:** ^1^ Department of Biology Emory University Atlanta Georgia 30322; ^2^ MRC Laboratory of Molecular Biology Cambridge Biomedical Campus Cambridge CB2 0QH United Kingdom

**Keywords:** RNA nuclear export, Nab2, polyadenylation, mRNA nuclear processing, poly(A) tail

## Abstract

The poly(A) RNA binding Zn finger ribonucleoprotein Nab2 functions to control the length of 3′ poly(A) tails in Saccharomyces cerevisiae as well as contributing to the integration of the nuclear export of mature mRNA with preceding steps in the nuclear phase of the gene expression pathway. Nab2 is constructed from an N‐terminal PWI‐fold domain, followed by QQQP and RGG motifs and then seven CCCH Zn fingers. The nuclear pore‐associated proteins Gfd1 and Mlp1 bind to opposite sides of the Nab2 N‐terminal domain and function in the nuclear export of mRNA, whereas the Zn fingers, especially fingers 5–7, bind to A‐rich regions of mature transcripts and function to regulate poly(A) tail length as well as mRNA compaction prior to nuclear export. Nab2 Zn fingers 5–7 have a defined spatial arrangement, with fingers 5 and 7 arranged on one side of the cluster and finger 6 on the other side. This spatial arrangement facilitates the dimerization of Nab2 when bound to adenine‐rich RNAs and regulates both the termination of 3′ polyadenylation and transcript compaction. Nab2 also functions to coordinate steps in the nuclear phase of the gene expression pathway, such as splicing and polyadenylation, with the generation of mature mRNA and its nuclear export. Nab2 orthologues in higher Eukaryotes have similar domain structures and play roles associated with the regulation of splicing and polyadenylation. Importantly, mutations in the gene encoding the human Nab2 orthologue ZC3H14 and cause intellectual disability.

## Introduction

In eukaryotes, the separation by the nuclear envelope of transcription from translation enables mRNAs to be modified by capping, splicing, and polyadenylation. These processing steps are mediated by a large number of different proteins that interact with transcripts as they pass through the nuclear phase of the gene expression pathway before they are finally exported to the cytoplasm through nuclear pores.^1^, ^2^, ^3^ In budding yeast, Nab2, which is the founding member of an evolutionarily conserved family of polyadenosine RNA binding Zn finger proteins,^4^ functions in regulating the length of poly(A) tails, compacting mature transcripts, and coordinating key nuclear RNA processing steps with RNA export (reviewed in Reference 5). *Saccharomyces cerevisiae* Nab2 (*Sc*Nab2) accompanies mature transcripts as they move through nuclear pores to the cytoplasm, after which *Sc*Nab2 is thought to be removed from the mRNA by the DEAD‐box RNA helicase, Dbp5^6^ that is located at the cytoplasmic face of the pore. *Sc*Nab2 is then recycled back to the nucleus through nuclear pores using the transport factor, karyopherin β2 (also termed Kap104 or transportin).^7^, ^8^ In addition, *Sc*Nab2 shows genetic interactions with the splicing machinery^9^ and also appears to function in mRNA quality control^10^ and Pol‐III transcription.^11^

In higher eukaryotes, the human Nab2 orthologue, ZC3H14, and *Drosophila* orthologue, *Dm*Nab2 are also required for proper poly(A) tail length control,^12^, ^13^ in addition to the well‐characterized poly(A) tail length regulator and nuclear poly(A) binding protein, PABPN1.^14^, ^15^ Importantly, mutations in the human *ZC3H14* gene have been linked to a nonsyndromic form of autosomal recessive intellectual disability,^12^, ^16^ linking Nab2/ZC3H14 to proper neuronal function. In strong support of a role for Nab2 in the brain, *Dm*Nab2 mutant flies exhibit impaired short‐term memory and defects in neuronal patterning in the learning and memory center (mushroom body) of the fly brain.^12^, ^17^ Notably, neuronal expression of human ZC3H14 in *DmNab2* mutant flies rescues function, indicating that ZC3H14 is a functional orthologue of *Dm*Nab2.^13^

## Molecular Architecture of Nab2 Family Members

**Figure 1:**
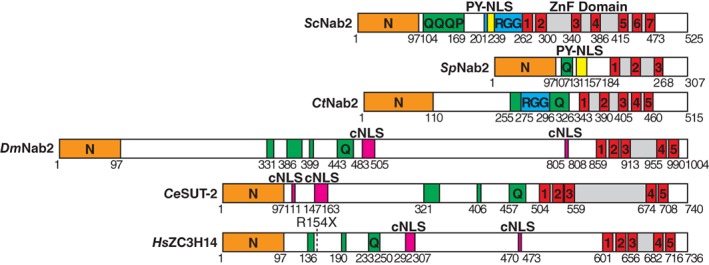
Members of the Nab2/ZC3H14 zinc finger polyadenosine RNA binding protein family have a similar domain architecture. Nab2 orthologues – S. cerevisiae Nab2 (*Sc*Nab2; Uniprot ID: P32505), *S. pombe* Nab2 (*Sp*Nab2; Uniprot ID: O13713), *C. thermotolerans* Nab2 (*Ct*Nab2; Uniprot ID: G0SCL7), Drosophila melanogaster Nab2 (*Dm*Nab2; Uniprot ID: Q9V3H9), Caenorhabditis elegans SUT‐2 (*Ce*SUT‐2; Uniprot ID: Q95XU6), and Homo sapiens (*Hs*ZC3H14; Uniprot ID: Q6PJT7) share a common domain structure based on a N‐terminal PWI‐fold domain (orange), which serves as a protein–protein interaction domain in *Sc*Nab2, followed by a Q‐rich region (green), and a C‐terminal Zn finger (ZnF) domain (gray) containing a series of Zn fingers (red), which bind to polyadenosine RNA. *Sc*Nab2 and *Ct*Nab2 contain an RGG domain (blue), which functions in karyopherin‐based nuclear import in *Sc*Nab2. In addition, *Sc*Nab2 contains a Pro‐Tyr nuclear localization signal (PY‐NLS) (yellow) and *Sp*Nab2 contains a predicted PY‐NLS that functions in nuclear import in *Sc*Nab2. Nab2 orthologues from higher Eukaryotes contain two predicted classical nuclear localization signals (cNLS) (magenta) that function in karyopherin‐based nuclear import. The nonsense mutation R154X identified in ZC3H14 in individuals with autosomal recessive intellectual disability is highlighted.

Members of the Nab2 protein family share a common architecture (Fig. 1) exemplified by the founding member *Sc*Nab2 in *S. cerevisiae. Sc*Nab2 contains an N‐terminal domain that has a Proline‐Tryptophan‐Isoleucine (PWI)‐like fold,^18^ followed by a Glutamine (Q)‐rich region of variable length, an Arginine‐Glycine (RGG) domain that functions as a nuclear targeting sequence in budding yeast, and finally a C‐terminal domain that contains seven tandem Cysteine‐Cysteine‐Cysteine‐Histidine (CCCH) Zn fingers that mediates high‐affinity binding to polyadenosine RNA.^4^, ^19^ These *Sc*Nab2 Zn fingers (ZnFs) are arranged into three groups: ZnF12; ZnF34; and ZnF567. The Nab2 orthologues in other species—*S. pombe* Nab2 (*Sp*Nab2); *C. thermophilum* Nab2 (*Ct*Nab2); *D. melanogaster* Nab2 (*Dm*Nab2); *C. elegans* SUT‐2 (*Ce*SUT‐2); *H. sapiens* ZC3H14 (*Hs*ZC3H14)—have a similar overall domain architecture (Fig. 1). Although the N‐terminal domain (PWI fold) and Zn finger domain are highly conserved in all Nab2 orthologues, the number of Zn fingers varies in Nab2 orthologues with *Sp*Nab2 having only three and human ZC3H14 and most other Nab2 orthologues having five (Fig. 1).

Although the steady‐state localization for the Nab2 family members that have been studied is nuclear,^4^, ^12^, ^19^, ^20^, ^21^, ^22^, ^23^ there is evidence that *Sc*Nab2 can shuttle into and out of the nucleus^24^, ^25^ and in higher eukaryotes Nab2/ZC3H14 can be detected in the cytoplasm of neuronal cells.^26^ As shown in Figure 1, the nuclear localization signals (NLSs) that target Nab2 family members to the nucleus vary between species. The RGG nuclear targeting signal within *Sc*Nab2 has been defined experimentally,^7^ as has a PY‐NLS motif in *Sc*Nab2.^27^ In higher eukaryotes, the RGG nuclear targeting domain is replaced by two predicted classical lysine‐rich nuclear localization signals (cNLSs).

Binding to polyadenosine RNA, a primary function of the Nab2 protein family, is mediated by the Zn finger motifs.^4^, ^19^, ^22^ Functional studies of the Zn fingers have been performed most extensively in *Sc*Nab2 and have been complemented with structural studies that provide insight into recognition of poly(A) RNA using *Ct*Nab2 and *Sc*Nab2.^28^, ^29^, ^30^ The grouping of the Zn fingers varies somewhat among species (Fig. 1). *Sc*Nab2 Zn fingers 5, 6, and 7 (ZnF567) are critical for function, and studies demonstrate that a *nab2* mutant lacking ZnF567 is not functional in budding yeast.^19^ Structure–function studies^28^, ^30^ have also defined key conserved residues in ZnF567 that are important for the proper function of *Sc*Nab2.

## Structure and Function of the Nab2 N‐Terminal Domain

**Figure 2:**
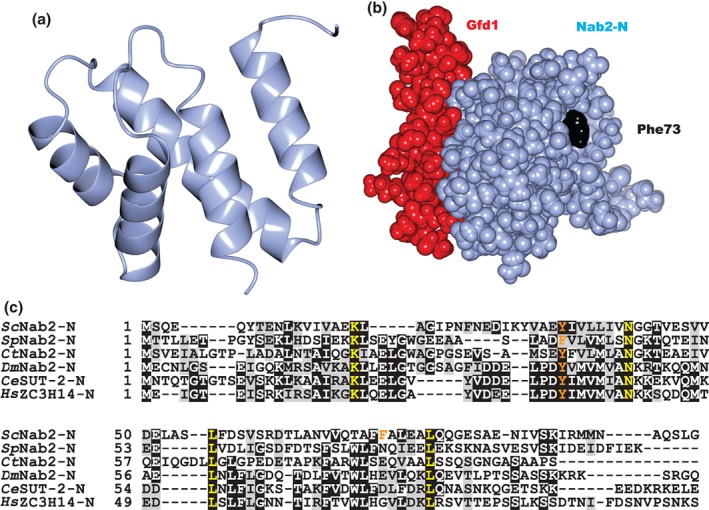
Structure of the S. cerevisiae Nab2 N‐terminal domain. (a) The Nab2 N‐terminal domain (Nab2‐N) has a PWI fold based on five α‐helices. (b) Interaction interfaces between Nab2‐N and the nuclear pore‐associated proteins Mlp1 (centred on Phe73 – Black) and Gfd1 (red) are on opposite sides of the Nab2‐N domain (based on PDB 3LCN). (c) The Nab2 N‐terminal domain is highly conserved between different Nab2 orthologues. Identical residues in all Nab2 orthologues are highlighted in yellow. *Sc*Nab2 residue Phe73 that is important for interaction with Mlp1, and *Sc*Nab2 residue Tyr34 that is important for interaction with Gfd1 are highlighted in orange. Identical residues (black) and similar residues (gray) are highlighted.

The crystal and solution structures of the *Sc*Nab2 N‐terminal domain (Nab2‐N)^31^, ^32^ showed that Nab2‐N has a PWI fold that is based on five α‐helices [Fig. 2(a)]. A budding yeast *nab2* mutant in which the N‐terminal domain has been deleted (*nab2‐ΔN*) exhibits severely impaired growth and nuclear accumulation of poly(A) RNA.^19^, ^33^ Moreover, bulk poly(A) tails are substantially longer in *nab2‐ΔN* cells, indicating that the N‐terminal domain might contribute to the control of poly(A) tail length.^34^ The *Sc*Nab2 N‐terminal domain interacts physically with both Mlp1, a component of the nuclear basket that is located on the nuclear face of nuclear pores,^31^, ^35^, ^36^ and Gfd1, which is thought to reinforce the function of the RNA export factor, Gle1.^31^, ^35^, ^36^ Mutagenesis studies have indicated that *Sc*Nab2 Phe73, which is located on a hydrophobic surface patch on the Nab2 N‐terminal domain [Fig. 2(b)], is important for the interaction with Mlp1, since *Sc*Nab2 F73A and F73D variants show impaired binding to Mlp1 in yeast lysates and *in vitro*.^31^, ^37^ The Mlp1‐Nab2 interaction could function to concentrate mature polyadenylated transcripts at the nuclear face of nuclear pores to facilitate their export to the cytoplasm^37^, ^38^ and also plays an important role in mRNP quality control.^39^

The *Sc*Nab2 N‐terminal domain also interacts with Gfd1,^32^ which is a multicopy suppressor of the *dbp5(rat8‐2)* RNA helicase mutant^40^ and the *gle1‐8* RNA export factor mutant.^41^ Crystallography, supported by solution NMR studies, showed that Gfd1 residues 126–150 form a helix when bound to the *Sc*Nab2 N‐terminal domain and identified a key contribution made by *Sc*Nab2 Tyr34, which is located on the opposite side of the N‐terminal domain to *Sc*Nab2 Phe73, which is important for Mlp1 binding^32^ [Fig. 2(b,c)]. Critically, a *nab2‐Y34A dbp5(rat8‐2)* double mutant shows a synthetic slow growth phenotype.^32^ Together, these results support the importance of the Nab2‐Gfd1 interaction for Dbp5 function, which is crucial for remodeling mRNPs following nuclear export *in vivo*.

Although the N‐terminal domain is conserved in Nab2 orthologues [Fig. 2(c)], there is currently little information regarding the function of this domain in higher eukaryotes. However, deletion of the N‐terminal domain of human ZC3H14 (ZC3H14‐N) does not impair its nuclear localization,^42^ consistent with the observation that the predicted cNLSs of ZC3H14 are C‐terminal of ZC3H14‐N (Fig. 1). The observation that *Sc*Nab2‐N interacts with the nuclear pore associated proteins, Mlp1 and Gfd1, suggests that Nab2‐N PWI domain could also function as a protein–protein interaction module in higher eukaryotes, although no partners have currently been identified.

## Structure of Nab2 Zn Fingers

The structures of several Nab2 Zn finger clusters have been established using both X‐ray crystallography and NMR.^28^, ^29^, ^30^, ^43^ In contrast to many other Zn finger proteins, all the Nab2 structures show that the Zn fingers interact with one another to varying extents and so have defined orientations to one another. *Sc*Nab2 ZnF567^28^, ^30^ and the corresponding ZnF345 from *Ct*Nab2^29^ form single structural units, in which the Zn fingers are arranged so that the first and third Zn finger are arranged on one side of the unit, with the middle Zn finger directed toward the opposite side. This 3‐dimensional spatial relationship between the Zn fingers precludes a single poly(A) RNA chain binding to all of them simultaneously. Similarly, NMR has demonstrated that *Sc*Nab2 ZnF1 and ZnF2 interact with one another as do *Sc*Nab2 ZnF3 and ZnF4 to form defined structural units.^43^

**Figure 3:**
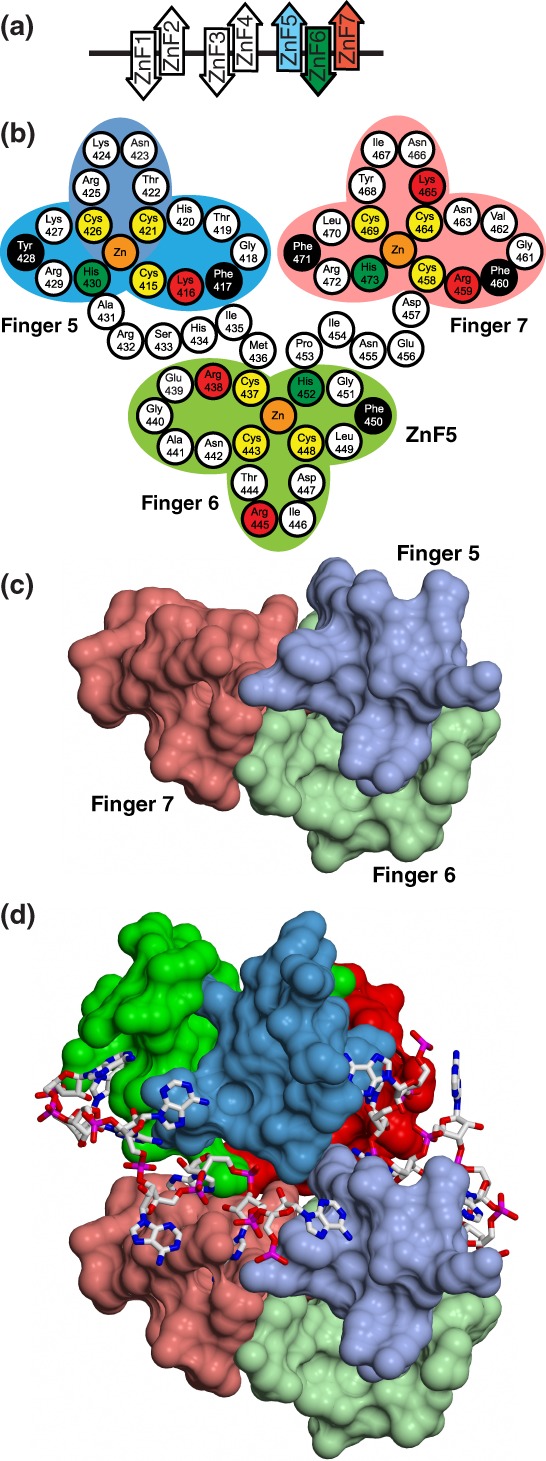
Nab2 Zn fingers. (a) Schematic illustration of the structures of the S. cerevisiae Nab2 Zn fingers determined by NMR (28, 43) and X‐ray crystallography.^29^, ^30^ The Zn fingers are clustered into three groups, and the fingers within each group have a defined orientation that influences their interaction with adenine‐rich RNA chains. (b) Arrangement of residues in *Sc*Nab2 Zn fingers 5–7. Key hydrophobic and basic residues that are important for binding to adenines (see also Fig. 4) are black and red, respectively. (c) Structure of *Sc*Nab2 Zn fingers 567. Zn finger 6 (green) projects on the opposite of the module to Zn fingers 5 (blue) and 7 (red). (d) Heterotetramer produced by A_11_G binding to *Sc*Nab2 Zn fingers 567 in which each RNA chain binds to both protein chains. Based on PDB 5L2L Panel (B) is derived from C. Brockmann et al. (2012) “Structural basis for polyadenosine‐RNA binding by Nab2 Zn fingers and its function in mRNA nuclear export.” *Structure*, **20**:1007–1018.

**Figure 4:**
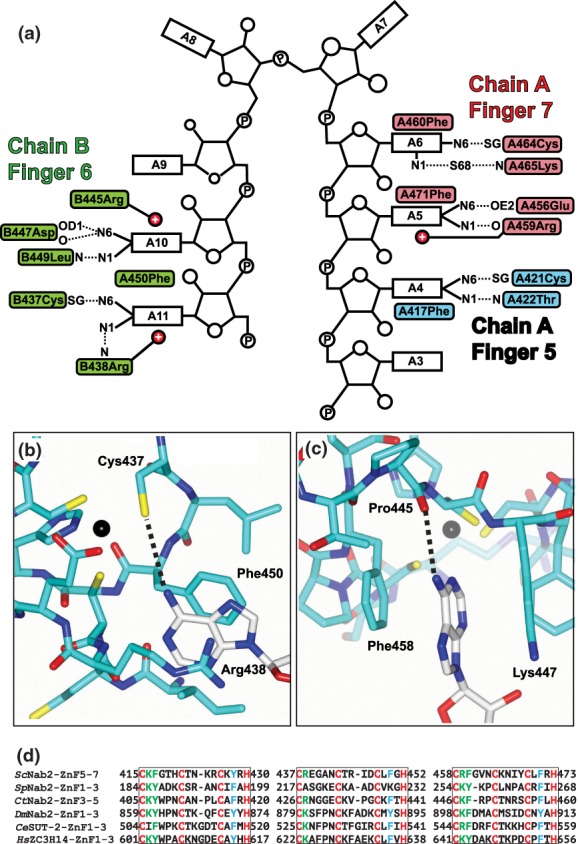
Interaction of *Sc*Nab2 Zn fingers 567 with adenine‐rich RNA. (a) Interactions between a single A_11_G RNA chain and the two Nab2 chains within a heterotetramer. Adenine rings are bound into surface clefts and are sandwiched between an aromatic residue on one side and a basic residue on the other that participates in a cation‐π interaction. The specificity of the interaction derives from H‐bonds formed by the adenine N6 and either a cysteine SG bound to Zn (b) or a main chain carbonyl (c). In each case, guanine cannot interact in this way because its O6 lacks hydrogen. Based on PDB 5L2L. (d) *Sc*Nab2 Zn fingers 567 are evolutionarily highly conserved between different Nab2 orthologues. *Sc*Nab2 ZnF567 are most similar to ZnF123 in most Nab2 orthologues and ZnF345 in *Ct*Nab2. Key conserved basic and aromatic residues (green) follow the first cysteine in the Zn fingers and conserved aromatic residues (blue) follow the third cysteine in Zn fingers. These conserved basic and aromatic residues interact specifically with polyadenosine RNA in S. cerevisiae and *C. thermophilum* Nab2. Panels (a), (b), and (c) reproduced from S. Aibara *et al. “*Structural basis for the dimerization of Nab2 generated by RNA binding provides insight into its contribution to both poly(A) tail length determination and transcript compaction in Saccharomyces cerevisiae.” *Nucleic Acids Res*. **45**:1529–1538 (2017) by permission of Oxford University Press. Available at DOI: 10.1093/nar/gkw1224.

In *Sc*Nab2 ZnF567, NMR chemical shift perturbations associated with binding either AMP or A_3_ identified a series of basic and aromatic residues associated with RNA binding^28^ [Fig. 3(b)]. These *Sc*Nab2 ZnF567 residues are strongly conserved between Nab2 orthologues [Fig. 4(d)] and *Sc*Nab2 variants in which these ZnF residues were substituted showed reduced affinity for A_9_ RNA.^28^ Although *nab2* ZnF567 mutants containing substitutions of these basic and aromatic residues had growth rates similar to wild‐type cells, the *nab2* ZnF567 mutants generated longer poly(A) tails *in vivo* and also showed genetic interactions with both Dbp5 and Yra1, consistent with their also influencing the generation of mature mRNPs.^28^ Moreover, in *S*cNab2, structural coherence between ZnF567 was lost in the *Sc*Nab2 RNA‐binding mutant, nab2‐C437S, in which a Ser was substituted for the first Zn‐coordinated Cys in ZnF6 [Fig. 3].^28^ Importantly, the *nab2‐C437S* yeast mutant exhibited cold‐sensitive growth and hyperadenylation of bulk poly(A) tails.^28^ Furthermore, combining the *nab2‐C437S* mutant with the *dbp5(rat8‐2)* RNA helicase mutant suppressed the growth defect of the *dbp5*(*rat8‐2*) mutant.^28^ Analysis of additional structure‐guided *nab2* ZnF mutants in combination with the *dbp5(rat8‐2)* mutant indicated that *dbp5(rat8‐2)* suppression by *nab2* ZnF mutants was more closely linked to hyperadenylation and suppression of mutant alleles of the nuclear RNA export adaptor, Yra1, than to the affinity of the mutant Nab2 for poly(A) RNA.^28^ Overall, these results indicate that, in addition to modulating poly(A) tail length, *Sc*Nab2 has an unanticipated function associated with generating export‐competent mRNPs, and that changes within ZnF567 lead to suboptimal assembly of mRNP export complexes that are more easily disassembled by Dbp5 upon reaching the cytoplasm.

## Structural Basis for the Interaction of the Zn Fingers with Poly(A) RNA

The crystal structures of *Sc*Nab2 ZnF567^30^ and *Ct*Nab2 ZnF345^29^ bound to A_11_G and A_8_ RNA, respectively, indicated the basis for selective binding of Nab2 Zn fingers to adenine and identified the importance of H‐bonds formed by adenine N6 (Figs. 3 and 4). In both structures of Nab2 Zn fingers bound to poly(A) RNA, the purine ring binds in a surface groove, where it stacks against an aromatic side chain on one side with a basic residue forming a cation‐π interaction on the other. These *Sc*Nab2 aromatic and basic Zn finger residues are strongly conserved in other Nab2 orthologues [Fig. 4(d)], and interactions between these *Sc*Nab2 residues and adenines were also seen in NMR studies.^28^ In addition, *Sc*Nab2 Zn finger variants in which these aromatic and basic residues were substituted showed reduced affinity for poly(A) RNA *in vitro* and generated longer bulk poly(A) tails *in vivo*.^28^ Specificity for the interaction is provided by a novel pattern of H‐bonds, most commonly between purine N6 and a Zn‐coordinated Cys residue or a main‐chain carbonyl, supplemented by H‐bonds between purine N7 and backbone amides. In both interactions involving adenine N6, the H‐bond formed either to a Cys SG thiol or a main‐chain carbonyl cannot be formed with guanine because its O6 does not have a donor hydrogen [Fig. 4(b,c)].

In the structures of both *Sc*Nab2 Zn fingers 567 and *Ct*Nab2 Zn fingers 345 complexed with poly(A) RNA, the spatial arrangement of the fingers precludes them from all binding to the same RNA chain, so that the first and third Zn fingers of the module were bound to one RNA chain, whereas the middle Zn finger was bound to a second RNA chain. Moreover, the crystal structure of *Sc*Nab2 ZnF567 complexed with A_11_G^30^ showed that binding RNA generated a distinctive heterotetramer that contained two protein chains and two RNA chains (Figs. 3(d) and 4(a)). *In vitro* binding studies^30^ demonstrated that this heterotetramer was also formed in solution between *Sc*Nab2 ZnF567 and either A_12_ or A_11_G, indicating that it was not a crystallization artifact and also that the 3′ terminal G was not necessary for its formation. Unusually, the dimerization of the *Sc*Nab2 protein chains was mediated almost entirely by each RNA chain binding to both protein chains and not by specific interactions between residues on the protein chains themselves, precluding the engineering of *Sc*Nab2 variants in which the Nab2‐Nab2 interaction was impaired. However, *Sc*Nab2 dimerization was impaired in Nab2 variants in which RNA binding was disturbed, with substitution of Phe450 in ZnF6, that is important for the interaction with the two adenines that bind ZnF6 (Fig. 4), showing the greatest decrease.^30^ Compared with other *Sc*Nab2 ZnF variants, *Sc*Nab2 F450A also showed one of the largest decreases in affinity for A_8_ RNA *in vitro* coupled with one of the largest increases in poly(A) tail length *in vivo*.^28^ Furthermore, the *Sc*Nab2 F450A variant exhibited some of the strongest genetic interactions with Yra1 and Dbp5.^28^ Combined, these data underscore the importance for Nab2 function of the dimerization induced by its binding to adenine‐rich RNA.

**Figure 5:**
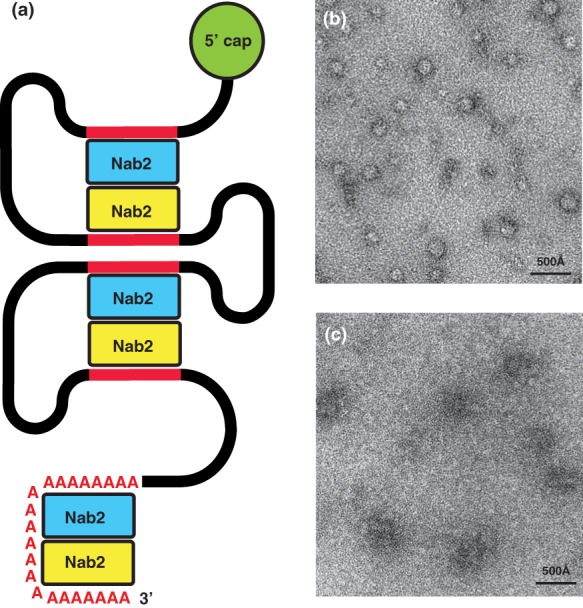
(a) The *Sc*Nab2 dimerization generated by its binding to two adenine‐rich regions of a transcript has the potential to both terminate 3′ polyadenylation and to facilitate compaction of the mRNP. (b) Electron micrograph of negatively stained *GAL1* RNA complexed with *Sc*Nab2 Zn fingers 567 shows that compact spherical particles were generated, whereas a more open, less condensed complex was obtained with the F460A variant (c) in which Nab2 dimerization is impaired. Panels (a), (b), and (c) are reproduced from S. Aibara *et al. “*Structural basis for the dimerization of Nab2 generated by RNA binding provides insight into its contribution to both poly(A) tail length determination and transcript compaction in Saccharomyces cerevisiae.” *Nucleic Acids Res*. **45**:1529–1538 (2017) by permission of Oxford University Press. Available at DOI: 10.1093/nar/gkw1224.

In the *Sc*Nab2 ZnF567‐A_11_G heterotetramer, not all the adenines interact directly with the Nab2 protein chains, suggesting that Nab2 could also form analogous dimers with RNA sequences that only contain adenines in key positions [Fig. 5(a)]. Binding to such A‐rich RNA sequences would be consistent with the observation that *Sc*Nab2 binds along the coding region of transcripts as well as to the poly(A) tail,^44^, ^45^ lending weight to the proposal^46^ that Nab2 could also have a function in mRNP compaction. Support for this hypothesis was provided by assessing the function of *Sc*Nab2 ZnF567 in mediating the compaction of *GAL1* transcripts *in vitro* [Fig. 5(b,c)]. Negatively stained electron micrographs indicated that wild‐type *Sc*Nab2 ZnF567, which were able to form dimers, resulted in much more compact complexes than those formed by the *Sc*Nab2 F450A variant, in which dimerization was impaired.^30^

## *Sc*Nab2 Regulation of Poly(A) Tail Length

**Figure 6:**
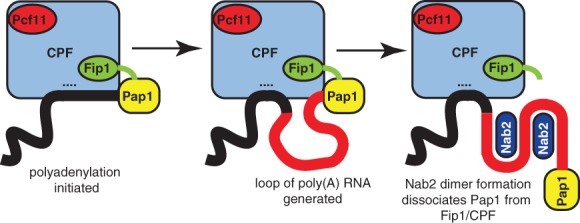
Function of *Sc*Nab2 in polyadenylation termination. Illustration of how the formation of an *Sc*Nab2 dimer could terminate polyadenylation by decreasing the flexibility of the poly(A) chain (red) so that poly(A) polymerase (Pap1—yellow) is dissociated from the cleavage and polyadenylation factor (CPF) so that it ceases to function processively and dissociates. The growing poly(A) chain (red) is held at its 5′ end by the transcript that is bound to the CPF (….) and at its 3′ end by Pap1 so that a flexible loop of poly(A) RNA is formed as polyadenylation progresses. When the poly(A) tail is sufficiently long, Nab2 binds and the resulting dimerization reduces the flexibility of the RNA so that Pap1 is forced to dissociate from the CPF and Fip1, after which it ceases to be processive and so dissociates from the poly(A) tail, terminating polyadenylation.

Polyadenylation is the final processing step in the nuclear phase of the gene expression pathway. In *S. cerevisiae*, the 3′‐end processing machinery comprises cleavage factors IA and 1B and the cleavage and polyadenylation factor (CPF), a complex including the riboendonuclease, Ysh1/Brr5, the poly(A) polymerase, Pap1, and the Pap1 regulation factor, Fip1.^47^ Rna15, associated with the scaffold protein, Rna14, and other CFIA subunits, Pcf11 and Clp1, positions the CPF to cleave the poly(A) site. After the cleavage reaction, poly(A) polymerase, Pap1, synthesizes a poly(A) tail of ~60–80 adenosines in a template‐independent manner. Because isolated Pap1 is an inefficient distributive enzyme, Pap1 processivity requires stimulation/regulation by factors that bind Pap1 and tether it to the RNA.^14^, ^47^, ^48^, ^49^ In mammals, the RRM‐containing nuclear poly(A) RNA binding protein, PABPN1, enhances the processivity of PAP and, in *S. cerevisiae*, the CPF component, Fip1, binds Pap1 directly and tethers it to CPF to stimulate/regulate Pap1 activity.^48^, ^49^ Figure 6 illustrates a possible mechanism by which Nab2 could control poly(A) tail length in which Nab2 binding to the growing poly(A) chain results in the dissociation of poly(A) polymerase Pap1 from the CPF, analogous to that generated by PABPN1 in higher eukaryotes.^5^, ^14^, ^50^, ^51^ As polyadenylation proceeds, a growing loop of poly(A) RNA is generated because the poly(A) tail is held both by the CPF and Pap1. This loop can be accommodated both by the inherent flexibility of the poly(A) RNA and by the flexibility of Fip1^49^ to which Pap1 is attached (Fig. 6). However, when the poly(A) tail becomes sufficiently long, a complex could be formed in which Nab2 could bind and generate a dimer by the RNA wrapping around two protein chains, analogous to the heterotetramer seen with ZnF567.^30^ Formation of this Nab2‐RNA complex could generate sufficient stiffening of the poly(A) RNA chain to result in the dissociation of Pap1 from the CPF and so terminate polyadenylation in a manner analogous to that proposed for PABPN1 in Metazoans.^14^, ^50^, ^51^ *S. cerevisiae* poly(A) tails have a length of ~60–80 nucleotides (reviewed by Reference 14), but it is not clear how many adenines are bound to a Nab2 dimer. A nuclease digestion study^52^ has indicated that Nab2 may bind ~25–30 nucleotides *in vitro*, but it is not clear whether this result reflects binding to a dimer or whether it might reflect digestion of the poly(A) RNA as it loops between Nab2 chains. Consequently, it is not clear whether generating a single Nab2 dimer is sufficient to terminate polyadenylation or whether instead it is necessary to form two dimers. Further work will be required to address this question.

In addition to terminating polyadenylation by dissociating Pap1 from the CPF, Nab2 may also contribute to regulating poly(A) tail length through interactions with the 3′‐5′ riboexonuclease exosome complex,^5^ which is known to trim back poly(A) tails. Binding of Nab2 to the poly(A) tail may protect a certain length of poly(A) RNA from digestion by the RNA exosome. This hypothesis is supported by the physical and genetic interactions seen between Nab2 and the nuclear exosome catalytic subunit, Rrp6, which has been shown to restrict the length of poly(A) tails.^44^, ^53^, ^54^ Furthermore, both recombinant *Sc*Nab2 and *Sp*Nab2 can protect poly(A) RNA from degradation by the RNA exosome *in vitro*.^20^, ^55^ Critically, in the absence of nuclear Nab2, nuclear mRNA is rapidly degraded by the RNA exosome.^56^ Moreover, if nuclear export of mature mRNPs is blocked, newly synthesized mRNA transcripts are quickly degraded and this has been proposed to be because of the reduced availability of Nab2, which is unavailable because it is bound to the older previously generated transcripts.^57^

Overall, extensive analyses of *Sc*Nab2 Zn finger variants has indicated that the *Sc*Nab2 ZnF567 interaction with polyadenosine RNA plays a central role in the regulation of poly(A) tail length and mRNA compaction in *S. cerevisiae*. In this context, the observation that the loss of *Dm*Nab2 in *Drosophila* and ZC3H14 in mice and humans results in longer poly(A) tails^12^, ^13^, ^55^ suggests that *Dm*Nab2 ZnF123 and ZC3H14 ZnF123, which are most similar to *Sc*Nab2 ZnF567 [Fig. 4(a)], could interact with polyadenosine RNA in a similar manner to *Sc*Nab2 to contribute to the control of poly(A) tail length. In the future, it will be informative to assess the functional consequences of specific *Dm*Nab2/ZC3H14 Zn finger variants, such as *Dm*Nab2 C879S and *Hs*ZC3H14 C622S in ZnF2 that are equivalent to *Sc*Nab2 C437S in ZnF6 [Fig. 3(b)].

## Interactions between Nab2 and the mRNA Nuclear Export Machinery

In addition to its function in controlling poly(A) tail length in *S. cerevisiae*, Nab2 also shows genetic interactions with components of the mRNA nuclear export machinery, such as Yra1, Sub2, and Mex67.^28^, ^37^, ^58^, ^59^ These data would be consistent with Nab2 having a role in signaling that polyadenylation had been completed and that the resultant mRNP was now suitable for export to the cytoplasm. Although *Sc*Nab2 may also interact directly with Mex67, one possible mechanism for signaling the completion of polyadenylation could be mediated through Pcf11 and the THO complex as a result of the dissociation of poly(A) polymerase Pap1 from the CPF complex.^58^, ^60^ In such a mechanism, Pcf11 could initiate the Sub2‐mediated mRNP remodeling that results in the dissociation of Yra1 and the generation of a Mex67:Mtr2‐bound export‐competent mature mRNP.

## Questions Outstanding

Although Nab2 dimerization following binding to the growing poly(A) tail could provide a mechanism by which Nab2 terminates polyadenylation in *S. cerevisiae*, direct evidence for such a dimer containing full‐length Nab2 or the way in which the protein chains are arranged in the Nab2 dimer has not yet been obtained either *in vitro* or *in vivo*. It is also unclear whether one or two Nab2 dimers are needed to terminate polyadenylation. Although CPF components, such as Pcf11, appear to participate in signaling the termination of polyadenylation to the mRNA export machinery (TREX/Yra1/Sub2/Mex67:Mtr2) that generates an export‐competent mRNP, precise details of the signaling pathway and how polyadenylation termination is transmitted to Pcf11 remain to be established. Similarly, although there is clearly crosstalk between Nab2/ZC3H14 and the splicing machinery, most notably involving genetic interactions between *Sc*Nab2 and the splicosome component, Mud2,^5^, ^9^ again the precise mechanism by which this information is transferred is unclear. In addition, although binding of Nab2 to nuclear basket component, Mlp1, appears to be important to localizing Nab2‐bound transcripts to nuclear pores to facilitate both export and processing, it is not clear how the Nab2‐containing mRNPs are then released from Mlp1 to allow mature mRNPs to be exported.

In summary, a combination of functional and structural studies has provided a wealth of insight into the way in which Nab2 regulates mRNA poly(A) tail length, contributes to mRNA compaction and the integration of nuclear steps in the gene expression pathway with nuclear export in *S. cerevisiae*. Nab2 orthologues also contribute to these functions in higher Eukaryotes, however, the greater complexity of these systems has made establishing precise molecular mechanisms and signaling pathways more difficult. The spatial arrangement of Nab2 Zn fingers facilitates dimerization when bound to adenine‐rich RNAs that is important for the termination of 3′ polyadenylation and transcript compaction, albeit the precise structure of the Nab2 dimers generated *in vivo* remains to be established. Overall, the wealth of information that has been generated about the structure of Nab2 and its interactions with other components of the nuclear gene expression machinery has laid the foundation for beginning to define the precise ways in which these pathways are coordinated and also provides insight into the contribution made by Nab2 orthologues to these processes in higher Eukaryotes.
